# Metastasis Suppressor NME1 Modulates Choice of Double-Strand Break Repair Pathways in Melanoma Cells by Enhancing Alternative NHEJ while Inhibiting NHEJ and HR

**DOI:** 10.3390/ijms21165896

**Published:** 2020-08-17

**Authors:** Gemma Puts, Stuart Jarrett, Mary Leonard, Nicolette Matsangos, Devin Snyder, Ying Wang, Richard Vincent, Benjamin Portney, Rachel Abbotts, Lena McLaughlin, Michal Zalzman, Feyruz Rassool, David Kaetzel

**Affiliations:** 1 Department of Biochemistry and Molecular Biology, School of Medicine, University of Maryland, Baltimore, MD 21201, USA; gemmasamanthacook@gmail.com (G.P.); kathryn.leonard@aacr.org (M.L.); nmatsangos@gmail.com (N.M.); devin.sharp@umaryland.edu (D.S.); yingoliva@gmail.com (Y.W.); richardlordvincent@gmail.com (R.V.); bportney@gmail.com (B.P.); MZalzman@som.umaryland.edu (M.Z.); 2 Department of Toxicology and Cancer Biology, College of Medicine, University of Kentucky, Lexington, KY 40536, USA; stuart.jarrett@uky.edu; 3 Department of Radiation Oncology, School of Medicine, University of Maryland, Baltimore, MD 21201, USA; RAbbotts@som.umaryland.edu (R.A.); ljmcl@umaryland.edu (L.M.); FRassool@som.umaryland.edu (F.R.); 4 University of Maryland Marlene and Stewart Greenebaum Comprehensive Cancer Center, Baltimore, MD 21201, USA; 5 Department of Otorhinolaryngology, School of Medicine, University of Maryland, Baltimore, MD 21201, USA

**Keywords:** DNA repair, cancer, melanoma, metastasis, DNA double strand break repair, non-homologous end-joining, homologous recombination, homing endonuclease

## Abstract

Reduced NME1 expression in melanoma cell lines, mouse models of melanoma, and melanoma specimens in human patients is associated with increased metastatic activity. Herein, we investigate the role of NME1 in repair of double-stranded breaks (DSBs) and choice of double-strand break repair (DSBR) pathways in melanoma cells. Using chromatin immunoprecipitation, NME1 was shown to be recruited rapidly and directly to DSBs generated by the homing endonuclease I-PpoI. NME1 was recruited to DSBs within 30 min, in concert with recruitment of ataxia-telangiectasia mutated (ATM) protein, an early step in DSBR complex formation, as well as loss of histone 2B. NME1 was detected up to 5 kb from the break site after DSB induction, suggesting a role in extending chromatin reorganization away from the repair site. shRNA-mediated silencing of NME1 expression led to increases in the homologous recombination (HR) and non-homologous end-joining (NHEJ) pathways of double-strand break repair (DSBR), and reduction in the low fidelity, alternative-NHEJ (A-NHEJ) pathway. These findings suggest low expression of NME1 drives DSBR towards higher fidelity pathways, conferring enhanced genomic stability necessary for rapid and error-free proliferation in invasive and metastatic cells. The novel mechanism highlighted in the current study appears likely to impact metastatic potential and therapy-resistance in advanced melanoma and other cancers.

## 1. Introduction

Metastasis suppressors are a unique class of genes (MSGs) that suppress metastatic potential of cancer cells without affecting their growth characteristics in vitro and in vivo [1]. *NME1* (previously termed NM23-H1, or nucleoside diphosphate kinase-A/NDPK-A) is an MSG prototype, exhibiting reduced expression and metastasis suppressor function in melanoma, breast carcinoma, and other human cancers [2]. *NME1* expression suppresses the motile and invasive activities of cancer cells, and attention has largely focused on its interactions with signaling cascades in the cytoplasmic and cell membrane compartments [3,4,5].

Our laboratory, and others, have shown that NME1 and its homolog NME2 are also DNA-binding proteins [6], with NME1 promoting repair of DNA damage induced by ultraviolet radiation [7,8]. NME1 possesses a nucleoside diphosphate kinase (NDPK) function that maintains proper equilibrium of nucleoside di- and tri-phosphates [9]. The NDPK function of NME1 delivers ATP and GTP directly to other protein complexes in a process termed “substrate channeling” [10,11], which could potentially also contribute to DNA polymerase activity in DNA replication and repair [12,13]. NME1 also exhibits 3′–5′ exonuclease activity against single-stranded DNA substrates in vitro [14]. 3′-5′ exonucleases are also required for proofreading and DNA repair [15]. In particular, repair of double-stranded breaks in DNA (DSBs) via homologous recombination (HR) pathways requires trimming of overhanging 3′-termini by 3′-5′ exonuclease such as MRE11 and this activity can sometimes also occur during non-homologous end joining (NHEJ) [16]. This suggests NME1 may augment or complement the activity of other 3′-5′ exonucleases in DSB repair (DSBR). Progression of cancer to lethal metastatic forms is driven by the accrual of genetic [17] and epigenetic [18] alterations. The reduced expression of NME1 would appear likely to confer genomic instability and acquisition of progression-driving alterations in DNA structure (e.g., point mutations, insertions/deletions, translocations). In this regard, we showed that point mutations disabling the 3′-5′ exonuclease activity of NME1 are associated with loss of metastasis suppressor function in melanoma cells [19].

HR is a high-fidelity pathway for DSBR operative during the S and G2 phases of the cell cycle that accesses the unaffected sister chromatid for correct sequence information during the repair process [20]. Classical or canonical NHEJ (C-NHEJ) is the predominant DSBR system in non-proliferating cells, particularly during periods in the cell cycle when the sister chromatid is inaccessible as a template. C-NHEJ relies upon an initial processing step to excise non-ligatable termini, followed by ligation of the modified ends. In NHEJ, the end-processing step and the lack of access to correct sequence information in the sister chromatid contribute to a lower fidelity of repair than that conferred by HR. Another NHEJ pathway is the so-called alternative NHEJ pathway (A-NHEJ) that is highly error-prone and regarded as a salvage or backup pathway activated when cells are overwhelmed with DNA damage or C-NHEJ or HR pathways fail [21,22,23]. A-NHEJ exploits longer regions of microhomology and co-opts various proteins that also function in HR, such as MRN factors, PARP-1, WRN and LIG1. A-NHEJ exhibits much lower fidelity than C-NHEJ and has been implicated in genomic instability during tumorigenesis [24]. We first reported that NME1 is recruited to DSBs in melanoma cell lines [25,26], and this has recently been confirmed [27]. NME1 has also shown to enhance total NHEJ activity in A549 (lung adenocarcinoma) [28], U2-OS (osteosarcoma) and HEK293 (embryonic kidney) cells but was reported to exert no impact on HR activity [27].

While primary melanoma is predominantly caused by accumulation of oncogenic mutations under conditions of genomic instability, progression to metastasis requires a return to genomic stability in order for proliferation to be both fast and free of errors [29]. This reversion to efficient DNA repair capacity has also been suggested to be responsible for therapy resistance of metastatic tumors. These considerations suggested the hypothesis that reduced NME1 expression in melanoma cells not only confers increased metastatic activity, but also enhanced genomic stability via downregulation of the low fidelity A-NHEJ pathway and upregulation of the higher fidelity HR and C-NHEJ pathways. Herein, we systematically examined the contribution of NME1 to total NHEJ, A-NHEJ and HR pathways of DSBR in melanoma cells. We report here that NME1 is recruited rapidly and directly to DSBs. We also show that in the specific setting of human melanoma cells, NME1 expression exerts a suppressive influence on the higher-fidelity HR and NHEJ pathways while enhancing the lower fidelity A-NHEJ pathway. Conversely, these studies indicate that low NME1 expression in melanoma cells results in reprogramming of DSBR activities to increase genomic stability. The effects of NME1 depletion on these three DSBR pathways were exerted in cell lines representing both early stage (WM35) and metastatic (WM164) melanoma, strongly suggesting the genome-stabilizing effect of NME1 appears early in melanoma progression. To our knowledge, this is the first demonstration that NME1 regulates A-NHEJ and HR activity. These observations support the hypothesis that loss of NME1 expression and attendant genetic stabilization provides an advantage for invasive and metastatic properties of tumor cells, as well as resistance to chemo- and radiotherapies.

## 2. Results

### 2.1. NME1 Is Recruited to DNA Repair Foci Following Induction of DSBs

**Figure 1:**
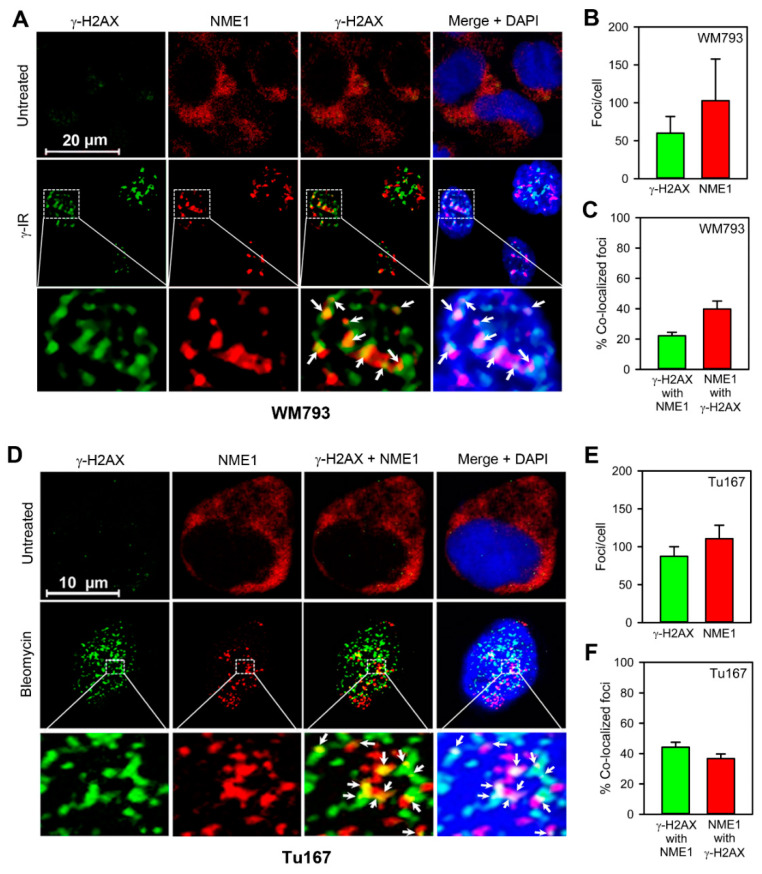
Induction of double-stranded DNA breaks (DSBs) induces nearly complete translocation of NME1 from the cytoplasmic compartment into DNA repair foci within the nucleus. (**A**) DSB induction in WM793 melanoma cells with γ-irradiation (γ-IR, 8 Gy) results in co-localization of NME1 with γ-H2AX foci. Foci were assessed at 1h post-induction. Arrows denote regions of colocalization between NME1 and γ-H2AX foci. (**B**) Quantification of γ-IR-induced γ-H2AX and NME1 foci in WM793 cells. (**C**) Co-localization of γ-IR -induced γ-H2AX and NME1 foci in WM793 cells. (**D**) DSB induction in Tu167 (head and neck) squamous cell carcinoma cells with bleomycin (2 h, 20 ng/mL) results in co-localization of NME1 with γ-H2AX foci. Methods for immunofluorescence are provided in SI. Images were captured using a Zeiss 510 laser confocal microscope. (**E**) Quantification of bleomycin-induced γ-H2AX (green) and NME1 (red) foci in Tu167 cells. (**F**) Co-localization of bleomycin-induced γ-H2AX and NME1 foci in Tu167 cells. Colocalization was calculated based on centers of mass-particles coincidence using the JACoP ImageJ plugin from the ImageJ software package [32]. Results in panels (**B**,**C**,**E**,**F**) are expressed as mean ± S.E.M (n = 6 fields per treatment group, with at least 5 cells per field) and were derived from three independent experiments.

To address the potential contribution of NME1 to DSBR, we first measured the association of NME1 with DSB-induced nuclear foci. In WM793 melanoma cells, induction of DSBs following γ-irradiation (γ-IR; 1 h, 8 Gy) resulted in induction of foci positive for the DSB biomarker γ-H2AX [30] (Figure 1A). While immunostaining of untreated WM793 cells with anti-NME1 antibody was diffuse and exclusively extra-nuclear, γ-IR treatment resulted in complete translocation of NME1 into nuclear foci. γ-H2AX-positive and NME1-positive foci were absent from nuclei in the absence of γ-IR treatment. In addition, foci were strongly colocalized (Figure 1B,C). Similarly, γ-H2AX-positive and NME1-positive foci were absent from nuclei of Tu167 (head-neck squamous cell carcinoma; HNSCC) cells prior to induction of DSBs, but were strongly induced following treatment with the DSB inducer bleomycin nuclear foci that stained positively for the DSB biomarker γ-H2AX (Figure 1D,E). As with WM793 cells and γ-IR treatment, bleomycin treatment of Tu167 cells resulted in complete translocation of NME1 into nuclear foci. Moreover, approximately γ-H2AX-positive foci and NME1-positive foci were largely colocalized (40%; Figure 1F). These concordant observations obtained in two cell lines of distinct cancer origins indicate induction of DSBs stimulates profound translocation of NME1 into the nucleus and recruitment of NME1 to focal structures within the nucleus that are associated with DSBR.

### 2.2. NME1 Is Recruited Directly to DSBs

**Figure 2:**
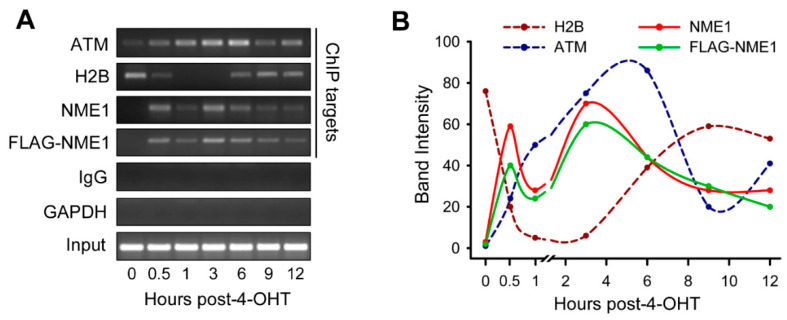
NME1 is recruited to double-strand breaks in DNA. (**A**) Analysis of chromatin immunoprecipitation (ChIP) DNA products by agarose gel electrophoresis and ethidium bromide staining. WM793 FLAG-NME1 cells were infected with HA-ER-I-PpoI retrovirus and treated with 1µM 4-OHT treatment for 15 min to induce translocation of I-PpoI into the nucleus. Cells were fixed at the indicated time points and ChIP performed using the indicated antibodies. Primers directed to the 28S rDNA I-PpoI target site were used, or directed to GAPDH as a negative control. Results shown are representative of two replicate experiments. (**B**) Quantification of bands in panel A using ImageJ for densitometry [32]. Band intensity values (*Y*-axis) corresponding to degree of relative brightness compared to background on a scale of 1–100.

Our laboratory has previously demonstrated in melanoma cell lines that NME1 expression enhances repair of ultraviolet light-induced DNA damage [8]. Having shown that NME1 is also recruited to DSBs conferred by γ-IR treatment in the melanoma cell line WM793, we elected to analyze the impacts of NME1 on DSBR in the specific setting of melanoma-derived cell lines. To determine whether NME1 is recruited directly to DNA lesions undergoing DSBR, DSBs were introduced at defined locations within the genome with the homing endonuclease I-PpoI, as described [31]. For initial studies, a clone of WM793 cells (793H1-FL8) that overexpresses FLAG peptide-tagged NME1 [8] was infected with a retroviral vector encoding an I-PpoI fusion protein (HA-ER-I-PpoI). The HA-ER-I-PpoI protein contains an estrogen receptor (ER)-derived sequence mediating tamoxifen-inducible translocation to the nucleus, and a hemagluttinin (HA)-based peptide tag for immunodetection. Treatment of I-PpoI retrovirus-infected cells with 4-hydroxytamoxifen (4-OHT) resulted in rapid recruitment of ataxia telangiectasia mutated kinase (ATM) to a known I-PpoI target site within the 28S rDNA locus, as detected by chromatin immunoprecipitation (ChIP; Figure 2A,B). ATM recruitment was seen within 30 min of DSB induction with 4-OHT and peaked at 3-6 h, with partial decay by 12 h. A trace amount of ATM was detected in association with the DSB site prior to 4-OHT treatment, likely attributable to low levels of I-PpoI-induced DSBs occurring after viral transduction but prior to the 4-OHT induction. Consistent with disruption of nucleosomes at the I-PpoI-induced DSB [31], histone 2B occupancy was lost within 0.5–1 h of 4-OHT treatment, and recovered to near-pretreatment levels by 6 h. The rapid 4-OHT-induced recruitment of ATM and loss of H2B, known early steps in DSBR, indicated that viral transduction and I-PpoI induction successfully generated DSBs at the 28S rDNA locus. NME1 was not detected by ChIP analysis at the 28S rDNA target site prior to DSB induction. However, 4-OHT treatment induced recruitment of NME1 to the DSB site within 0.5 h, which peaked at 3 h post-DSB induction and decayed to near-pretreatment levels by 12 h. Identical results were obtained with ChIP using anti-FLAG antibody directed to the overexpressed FLAG-tagged NME1 protein. While a subtle decrease in NME1 occupancy was apparent at 1 h post-induction, suggesting a possible biphasic response, this was not seen in other experiments. Together, these findings indicate that NME1 is recruited directly and rapidly to sites of DSBR, with kinetics of accumulation and decay slightly more rapidly than those of an early DSBR mediator, ATM.

**Figure 3:**
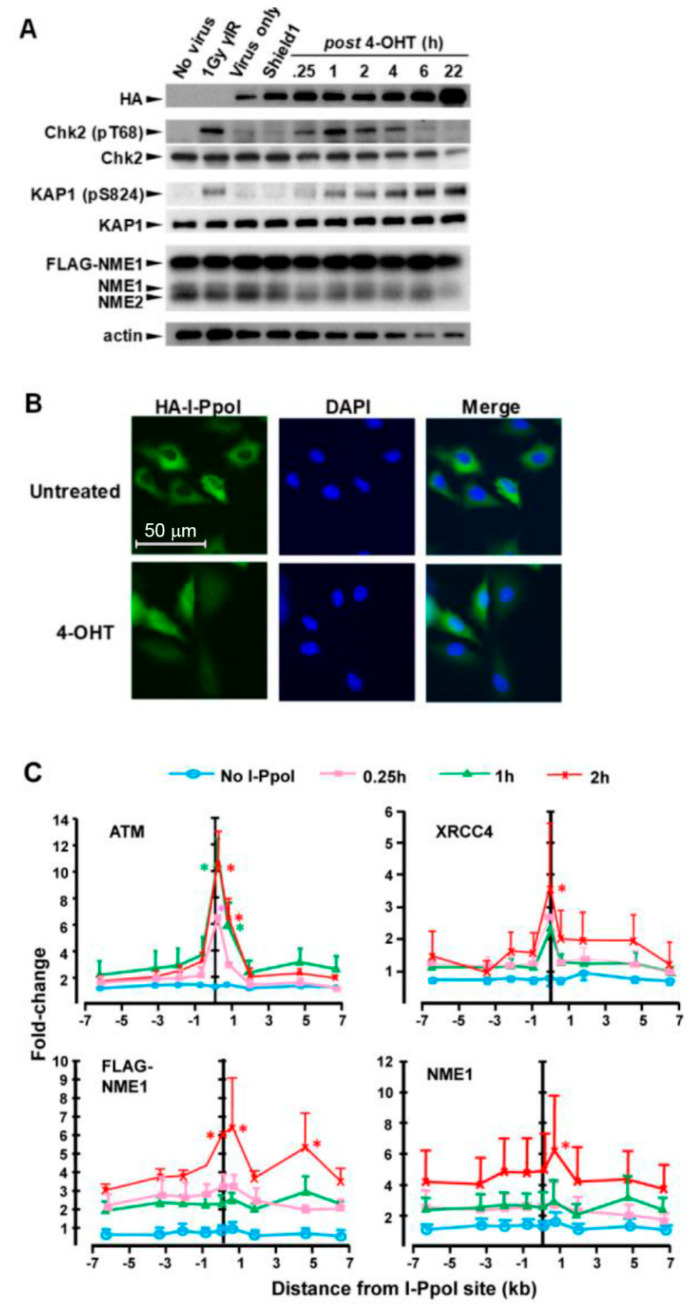
NME1 is recruited rapidly to DSB sites and adjacent regions of chromatin. (**A**) WM793-FLAG-NME1 cells were infected with a lentivirus (HA-ER-ddI-PpoI) harboring an HA-ER-IPpoI cassette tagged with a destabilization domain (dd) [34]. Infected cells were serum-starved for 24 h to enrich for cells in the G1 phase of the cell cycle, followed by treatment with Shield-1 to promote cytoplasmic accumulation of I-PpoI protein. Cells were then treated for 15 min with 4-OHT (1 mM) to initiate nuclear translocation and DSB-inducing activity of I-PpoI. Immunoblot analysis was conducted with the indicated antibodies on cell lysates obtained at the indicated times after 4-OHT treatment. (**B**) 4-OHT induces nuclear translocation of I-PpoI-ER-DD-HA fusion protein. (**C**) Cells were fixed at the indicated time points after I-PpoI induction and ChIP performed with the indicated antibodies. qPCR was conducted with nine different primer sets directed to amplicons spanning the 28S RNA target site (+/−6 kb). 1Gy of γ-IR was used as a positive control for response to DSB induction. Data points represent the mean + SEM of three independent experiments. Asterisks denote data points that are significantly different from untreated at time point of corresponding color at specific primer set, or in legend across all primers. *p* ≤ 0.05.

To examine spatio-temporal aspects of NME1 recruitment to DSBs, ChIP was conducted with a series of PCR amplicons spanning 6 kb upstream and downstream of the I-PpoI-induced break site at the 28S rDNA locus. For this study, a lentiviral vector was used to drive expression of a modified I-PpoI fusion protein (I-PpoI-ER-DD-HA) containing a destabilization domain (DD), reported previously to provide tight temporal control of I-PpoI induction [33]. Infection with I-PpoI-ER-DD-HA vector alone elicited a low level of protein expression, as detected by immunoblot with anti-HA antibody (Figure 3A). Sequential treatments with the DD inhibitor Shield-1 (1 h) and 4-OHT (15 min) resulted in further expression of the fusion protein, which persisted throughout a 22-h time course. 4-OHT-induced translocation of the I-PpoI fusion protein to the nucleus was verified by immunofluorescent staining (Figure 3B). 4-OHT inducible translocation of I-PpoI and DSB induction was further demonstrated by the rapid and transient phosphorylation of Chk2, an ATM substrate that governs checkpoint responses following DNA damage [34]. Chk2 activation was detected within 15 min of 4-OHT treatment, and achieved peak levels by 1 h that were equivalent to those obtained with γ-irradiation (γ-IR; 1 Gy, 1 h). I-PpoI-generated DSBs also induced phosphorylation-mediated activation of KAP-1, another ATM substrate and mediator of DSBR [35], with activation detected within 15 min and persisting over the 22-h time course. While the DD/Shield-1 system did not provide tight control of overall I-PpoI expression in the 793-FL8 cell line, translocation and ensuing DSB generation was inducible by the combined Shield-1 and 4-OHT treatments and, thus, was considered suitable for a spatiotemporal analysis of NME1 recruitment. ATM was recruited in a time-dependent fashion to DSBs, with a peak at 1 h post-induction. In similar fashion, the NHEJ mediator, XRCC4, was recruited rapidly to the DSB with a slightly later peak at 2 h. Both ATM and XRCC4 were localized almost entirely to the DSB site, with occupancy decreasing sharply with distance from the breakage. ChIP analysis with anti-FLAG and anti-NME1 antibodies again demonstrated I-PpoI-induced recruitment of NME1 to the DSB site, with peak levels observed at 2 h post-induction. Significant enrichment of NME1 was observed at the DSB, but significant occupancy was also detected 5 kb downstream with anti-FLAG antibody (Figure 3C). 

### 2.3. NME1 Downregulates Non-Homologous End Joining (NHEJ) and Homologous Recombination (HR), while Upregulating Alternative NHEJ (A-NHEJ)

To assess potential impacts of NME1 on the efficiency of NHEJ, A-NHEJ and HR pathways of double strand break repair, NME1 expression was silenced with shRNA in conjunction with intra-chromosomal repair assays selective for each pathway, as described [36,37,38]. Clones containing GFP substrates for the three repair assays (EJ5-GFP, NHEJ; EJ2-GFP, A-NHEJ; DR-GFP, HR) were generated by stable transfection of the human melanoma cell lines WM35 and WM164, as outlined in Materials and Methods.

These two parental cell lines were chosen on the basis of their origins from early (radial growth phase; RGP) and metastatic stages of melanoma progression, respectively. The EJ5-GFP substrate contains a constitutive promoter (phosphoglycerate kinase, or pgk) upstream of an I-SceI-flanked puro gene, followed by a GFP-coding cassette, and measures total NHEJ activity. The GFP sequence is converted to an NHEJ substrate by transient expression of I-SceI, which excises the puro gene to leave 4 bp of 3′-hydroxyl overhanging bases at both termini [36]. NHEJ-mediated repair of the 3′ overhangs is manifested as pgk-driven expression of GFP. A-NHEJ-mediated repair of I-SceI-induced double-stranded DNA breaks in the EJ2-GFP substrate results most often in a 35-nucleotide deletion with microhomology at the repair site when GFP expression is reconstituted [36]. Double-stranded DNA breaks induced with I-SceI in the DR-GFP sequence is repaired by HR, which uses a downstream GFP sequence as template for homology-directed recombination and recovery of GFP fluorescence [39].

**Figure 4:**
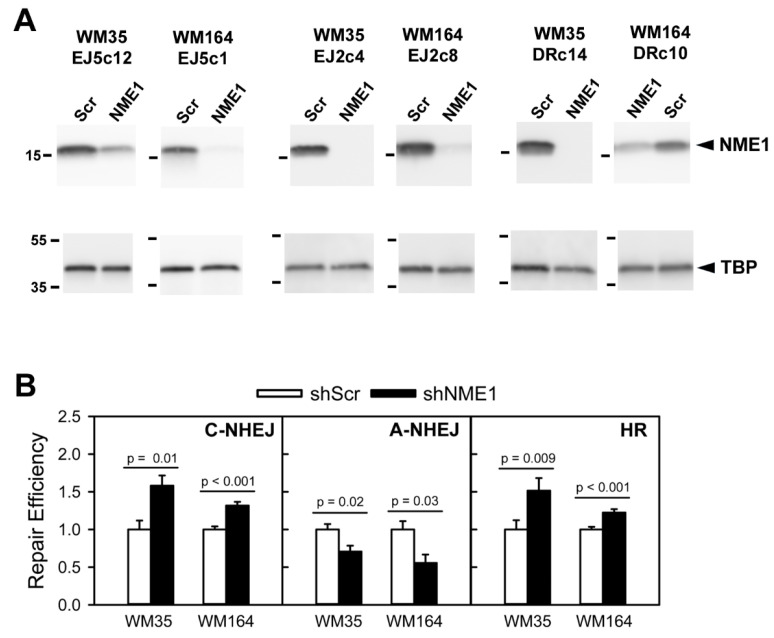
NME1 suppresses non-homologous end-joining (NHEJ) and homologous recombination (HR) in melanoma cells, while upregulating alternative NHEJ (A-NHEJ). Intra-chromosomal assays for NHEJ, HR and NHEJ were conducted in the human melanoma cell lines WM35 and WM164, with individual clones harboring one of the following stably integrated repair cassettes: EJ5-GFP (NHEJ), EJ2-GFP (A-NHEJ), or DR-GFP (HR). Cells were infected for 24 h with lentivirus encoding either a scrambled shRNA sequence (Scr) or an shRNA directed to NME1 (NME1), followed by induction of double-stranded DNA breaks by transient transfection with a plasmid encoding the homing endonuclease I-SceI. Repair activity (% GFP-positive cells) was measured 72 h post-transfection using flow cytometry (>10,000 cells analyzed per condition). (**A**) Validation of shRNA-mediated silencing of NME1 in melanoma cell lines. Immunoblot analysis of NME1 (upper panels) and TATA-binding protein (TBP, lower panels) expression in the indicated clones was conducted 96 h after infection with lentiviruses expressing either scrambled shRNA or shRNA directed to NME1. Cells for immunoblot analysis were infected in parallel with those used in the repair assays of panel B. Mobilities of NME1 and TBP are identified at the far right. (**B**) Differential regulation of NHEJ, A-NHEJ and HR by NME1. Data are represented as mean repair activity normalized for each clone against cells treated with shScr-expressing lentivirus. NHEJ assays were conducted in clones WM35EJ5c12 and WM164EJ5c1 (N = 8 from two replicate experiments for both clones), A-NHEJ assays in clones WM35EJ2c4 (N = 12 from 3 replicate experiments) and WM164EJ2c8 (N = 4), and HR assays in clones WM35DRc14 (N = 12 from three replicate experiments) and WM164DR10 (N = 16 from 4 replicate experiments). All experiments were conducted with quadruplicate wells for each treatment group. Detailed methods for generation of stably transfected clones, intra-chromosomal assays of NHEJ, A-NHEJ and HR activity, and statistical analyses are provided in Experimental Procedures. Original full-length images of scanned immunoblot membranes in panel A are shown in Figure S1.

Expression of shRNA directed to NME1 (shNME1) strongly silenced expression of NME1 protein in all clones (60–99%) relative to a scrambled version of the shNME1 sequence (Figure 4A). In contrast, expression of a negative control (TATA-binding protein; TBP) was unaffected by shNME1 expression across all clones. WM35- and WM164-derived clones harboring the EJ5-GFP cassette for assessment of NHEJ activity (WM35 EJ5c12 and WM164 EJ5c1) both exhibited highly significant increases in %GFP-positive cells following expression of the shNME1 sequence and induction of double-stranded breaks with I-SceI (Figure 4B). In contrast, expression of shNME1 suppressed A-NHEJ activity in WM35- and WM164-derived clones containing the EJ2-GFP cassette (WM35 EJ2c4 and WM164 EJ2c8). Finally, shNME1 expression resulted in highly significant increases in HR activity in WM35- and WM164-derived clones (WM35 DRc14 and WM164 DRc10) containing the DR-GFP cassette. Identical results were obtained across all DSBR pathways in clones derived from both parental WM35 and WM164 cell lines, suggesting the impact of NME1 is exerted across early and late stages of melanoma progression. Together, these findings indicate that NME1 suppresses the higher-fidelity HR and NHEJ pathways of double-strand break repair while enhancing low-fidelity A-NHEJ in cultured human melanoma cell lines.

## 3. Discussion

NME1 is a metastasis suppressor gene (MSG) that has provided valuable insights into mechanisms regulating cancer progression. While early studies focused on interactions of NME1 with motility-driving pathways in the intracellular and membrane compartments of the cell, critical roles have been identified in the nucleus as well. Our laboratory and others demonstrated that NME1 exhibits DNA-binding activity in vitro [6,40], suggesting the potential for direct interactions with the genome. In particular, recombinant human NME1 exerts a 3′–5′ exonuclease activity on single-stranded DNA templates [14], suggesting a role in DNA replication and/or repair [15]. Moreover, cellular expression of NME1 enhances efficiency and fidelity of nucleotide excision-mediated repair (NER) of UV-induced DNA lesions [7,8], although those studies did not address whether NME1 participates directly or indirectly (e.g., regulating signaling pathways, expression of repair genes) in the repair process.

The ChIP technique has been optimized to strongly favor covalent crosslinking of DNA-protein complexes and not protein–protein interactions [41]. Thus, ChIP was considered a powerful approach for assessing the extent to which NME1 protein is recruited not only to DSB lesions, but importantly the degree of its direct physical interactions with DNA at these sites. Although the possibility of crosslinking of protein–protein interactions cannot be completely excluded, the known DNA binding activity of NME1 in vitro together with our current studies provide compelling evidence for direct binding of NME1 to DSBs and nearby genomic DNA. NME1 possesses two enzymatic activities that could contribute to DSBR, its NDPK activity and a 3′-5′ exonuclease function [40].

In contrast with NER, both HR and NHEJ require 3′–5′ exonuclease activity to trim overhanging 3′-termini. MRE11 is a component of the MRN (MRE11-RAD50-NBS1) complex that can provide this function [42,43]. Nevertheless, it is unclear whether MRE11 is the only 3′–5′ exonuclease involved in DSBR. Some studies suggest its 3′–5′ exonuclease activity is most relevant to the HR pathway [44], while others have demonstrated that MRE11-dependent resection is dependent on the specific structures present at DSB termini [45]. This raises the potential for contributions by other autonomous 3′–5′ exonucleases (e.g., NME1) to repair of DSBs left unaddressed by MRE11. A point mutant form of NME1 defective in 3′–5′ exonuclease activity lacks the NHEJ-enhancing activity of the wild-type protein [27] suggesting a functional contribution of NME1 to 3′-end trimming in NHEJ, although this has yet to be demonstrated directly.

In addition to its 3′–5′ exonuclease activity, the NDPK activity of NME1 has been proposed to work in concert with ribonucleotide reductase (RNR) to enhance delivery of dNTPs to relevant DNA polymerases involved in DNA repair [13]. Both NHEJ and HR require DNA polymerase fill-in steps that could be facilitated by “channeling” of dNTPs by the NDPK/RNR complex. While NME1 recruitment is strongest at the DSB site itself, it is also recruited to a DNA sequence relatively distant from the DSB (Figure 3*C*). This is analogous to recruitment of γ-H2AX to broad regions flanking DSBs and other forms of DNA damage, and suggests NME1 contributes to the larger-scale chromatin reorganization required for physical access by components of the DSBR response. Consistent with a chromatin-modifying activity was our observation that the rapid NME1 recruitment to I-PpoI-induced DSBs was associated temporally with loss of histone 2B at the break site. In this regard, the NDPK/RNR complex also contains the histone acetylase Tip60, which is rapidly recruited to sites of DNA damage and indeed confers chromatin decondensation required for recruitment of DSBR enzymes and DSBR activity [46]. Of additional relevance are our recent observations in melanoma cells that NME1 induces transcription of three genes (*ALDOC*, *ITGB1* and *ITGB3*); this transcription-regulating activity is associated with both direct binding of NME1 and significant alterations in chromatin structure in the respective promoter regions [47,48].

A recent report shows that NME1 is required for NHEJ activity in osteosarcoma U2OS cells (27). In contrast, our results obtained with melanoma cells show that NME1 depletion promotes both NHEJ and HR activity, but is required for the more highly error-prone A-NHEJ. The apparent genome-destabilizing activity of NME1 identified in the current study would appear initially to be at odds with its metastasis suppressor function and the data of Xue et al. [27]. While the reason for these differences is not known, metastatic melanoma and relapse have been shown to be associated with upregulation of genes involved with multiple DNA repair pathways including those associated with DSBR [49,50,51,52]. This suggests metastatic cells such as those expressing reduced levels of the metastasis suppressor NME1 require a return to genomic stability in order for proliferation to be both rapid and error-free [29]. This reversion to efficient DNA repair capacity has also been suggested to be responsible for therapy resistance of metastatic tumors. That NME1 is well-established as a suppressor of metastasis in multiple cancers strongly suggests the broad relevance of its DNA repair functions to malignant progression. Our study does not exclude the possibility that NME1 may have a net genome-stabilizing effect through interactions with HR, NHEJ and A-NHEJ pathways in other cancers or in non-cancer settings. In summary, the ability of NME1 to directly regulate multiple DSBR pathways represents a novel molecular mechanism likely to impact metastatic potential and resistance to therapy in advanced melanoma and possibly other cancers as well.

## 4. Materials and Methods

### 4.1. Cell Culture and Chemicals

Unless otherwise specified, reagents were obtained from MilliporeSigma (Burlington, MA, USA). The melanoma cell lines WM35, WM164, WM793 were provided by M. Herlyn (Wistar Inst., Philadelphia, PA, USA). These cell lines and variants derived from them were cultured in MCDB153 medium supplemented with 2% FBS (Invitrogen, Carlsbad, CA, USA), 2.5 µg/mL insulin solution and 2 mM CaCl_2_ (Tu2%). The WM35 line was originally derived from a melanoma in radial growth phase (RGP), WM793 was isolated from a vertical growth phase (VGP) melanoma, and WM164 was derived from a metastatic lesion. WM793 clones expressing FLAG-NME1 or FLAG only (“empty vector” or FLAG only) were previously described [8,31]. The head and neck squamous cell carcinoma (HSSCC) cell line Tu167 was obtained from the University of Texas MD Anderson Cancer Center (Houston, TX, USA), and was cultured in complete Dulbecco’s Modified Eagle Medium (Invitrogen, high glucose) supplemented with 10% FBS (R&D Systems, Minneapolis, MN, USA), 2 mM Glutamax (Gibco, Carlsbad, CA, USA), penicillin (100 U/mL) and streptomycin (100 μg/mL).

### 4.2. Double-Strand Break Induction with γ-Irradiation and Bleomycin and Quantitation of DNA Repair Foci

For induction of DNA repair foci using γ-irradiation (γ-IR), cells in 8-well chamber slides were exposed to 8 Gy of γ-IR using a Nordion Gammacell 3000 Irradiator (Best Theratronics, Ottawa, ON, Canada). The 8 Gy dosage was optimized to provide a strong DNA damage response without induction of cell death. For DSB induction using bleomycin, cells were plated for 24 h on sterilized coverslips in 24-well plates. Cells were treated in triplicate for 2 h in complete DMEM containing 200 ng/mL bleomycin (MilliporeSigma) prior to analysis.

Following treatment, cells were washed twice with PBS and fixed in 4% PFA in DPBS for 10 min at room temperature. Cells were permeabilized with 0.25% NP-40 for 10 min. Cells were blocked for 10 min at room temperature in 1% BSA, 10% FBS, and 0.2% saponin, and incubated overnight at 4 °C with the primary antibodies in blocking solution: anti-γ-H2AX (1:1000), NME1 (1:1000). As additional negative controls, cells stained without primary antibody were used as well as the untreated isogenic cells. The bound antibodies were visualized with a fluorescent Alexa568 or Alexa488 secondary antibodies (Invitrogen) and nuclei were visualized with DAPI (Roche, Basel, Switzerland) staining for 10 min at room temperature. Coverslips were mounted and cells were visualized by Zeiss 510-confocal microscope followed by co-localization study performed by ImageJ software^1^ using the JACoP plugin [32]. Colocalization of NME with γ-H2AX was detected based on centers of mass-particles coincidence.

### 4.3. Lentivirus Production and Transduction

Lentiviral supernatants were produced for inducing expression of I-PpoI and NME1 in melanoma cell lines, using expression vectors pCL20C-ddIPpoI (Addgene, Cambridge, MA, USA) and pLenti-CMV-rtTA3G-Blast (R980-M38-658), respectively. From cell line 293T, cells were seeded on plates pre-coated with poly-l-lysine (MilliporeSigma). After 24 h, 293T cells at 50–70% confluency were transfected with 6:6:1 expression vector: pCD/NL-BH*DDD packaging vector: pCMV-VSVG envelope vector using Fugene 6 transfection reagent (Promega, Madison, WI, USA), 3:1 Fugene: total DNA. After 24 h, transfected cells were rinsed once with phosphate-buffered saline (PBS; Gibco) followed by addition of lentivirus dilution medium, consisting of Dulbecco’s modified Eagle’s medium (DMEM, high glucose; Gibco) supplemented with 10% charcoal:dextran stripped fetal bovine serum (FBS; GeminiBioo, Broderick, CA, USA) and 25 mM HEPES (Gibco). Supernatants were collected every 12 h, pooled, centrifuged at 500× *g* for 5 min at 4 °C and filtered on a 0.45-µm filter to remove cells and debris. Aliquots were stored at −80 °C. For expression of I-PpoI-ER-DD-HA, melanoma cells were infected at 70–80% confluency with a mixture of 25% virus supernatant and serum-free culture media containing 8 µg/mL polybrene (AmericanBio, Canton, MA, USA). Twenty-four hours after infection, infected cells were rinsed once with PBS, followed by another application of the 25% virus supernatant-containing medium. After 24 h, cells were treated with 1 µM AquaShield-1 (Cheminpharma, Branford, CT, USA) for 3 h, followed by 1 µM 4-hydroxytamoxifen (4-OHT; MilliporeSigma) for 15 min, then rinsed four times with PBS. Media was then replaced with serum-free medium and returned to 37 °C for the indicated periods of time. Lentiviruses expressing shRNAs for silencing of NME1 expression (shNME1b, 5′-tccgccttgttggtctgaaat-3′) and a scrambled version of the sequence (shScr) were produced as described [31]. In that study, the shNME1b sequence strongly silenced NME1 expression in multiple melanoma cell lines with minimal effect on the highly homologous NME2 isoform. Sequences of PCR primers and shRNAs are supplied in Table S1.

### 4.4. Immunoblotting

Cells were collected by cell dissociation buffer (HBSS; Gibco), lysed using RIPA buffer (10mM Tris-HCl pH7.5 (Fisher Scientific), 1 mM EDTA, 0.5 mM EGTA, 1% Triton X-100, 0.1% sodium deoxycholate, 140 mM sodium chloride (MilliporeSigma), 0.1% SDS (Quality Biological, Gaithersburg, MD, USA), and sheared by passing through a 25 gauge needle 10 times. After rotation at 4 °C for at least 45 min, protein concentrations of supernatants were assayed using DC Lowry (Bio-Rad, Hercules, CA, USA) and a 5–10-µg run on a Criterion AnykD Tris-HCl SDS-PAGE gel (Bio-Rad). Proteins were transferred to nitrocellulose, blocked in 5% milk, or 1% BSA for antibodies against phosphorylated proteins, diluted in tris-buffered saline containing 0.1% tween-20 (TBS-T) for 1 h, and incubated with primary antibody in 5% milk or 1% BSA overnight at 4 °C. Primary antibodies were used against: ATM (1:1000, Calbiochem, San Diego, CA, USA); p-ATM (1:1000, Santa Cruz Biotechnology, Santa Cruz, CA, USA); p-Chk2, Chk2, KAP1, histone H3 (1:2000, Cell Signaling Technology, Danvers, MA, USA); NME1 (1:2000, BD Biosciences, Ann Arbor, MI, USA); p-KAP1 (1:2000, Bethyl Laboratories Montgomery, TX, USA); HA (1:2000, Covance); γH2AX (1:2000), actin (1:1000), TATA box binding protein (1:500, MilliporeSigma); Rad50, XRCC4 (1:2000, Abcam, Cambridge, UK). Membranes were washed three times for 10 min in TBS-T, incubated with secondary antibody (goat anti-rabbit or goat anti-mouse IgG; GE Life Sciences, Marlborough, MA, USA) for 1 h, and washed with TBS-T again, three times for 10 min, before exposure to Prime ECL (GE Life Sciences).

### 4.5. Chromatin Immunoprecipitation (ChIP) and Quantitative Real-Time PCR (qPCR)

ChIP was completed using the ChIP-IT Express kit (Active Motif, Carlsbad, CA, USA), replacing the beads with ChIP-Grade Protein G Magnetic Beads (Cell Signaling Technology). Antibodies (5 µg) were used against ATM (Calbiochem), FLAG (MilliporeSigma), NME1 (Cell Signaling Technology) and XRCC4 (Abcam). The resulting DNA was purified using the QIAQuick PCR Purification Kit (Qiagen, Germantown, MD, USA), and 1 µL of each sample was used for qPCR, in quadruplicate, using *Power* SYBR Green Master Mix (Applied Biosystems, Foster City, CA, USA) with each of the primer sets detailed in supplemental Table S1.

### 4.6. Intra-Chromosomal Reporter Assays for Double-Strand Break Repair

To generate clones harboring GFP cassettes for intra-chromosomal reporter assays of double-strand break repair, the human melanoma cell lines WM35 and WM164 were transfected stably with one of the following plasmids: pDRGFP (a gift of Maria Jasin; Addgene plasmid #26475), EJ2GFP-puro or pimEJ5GFP (gifts of Jeremy Stark; Addgene plasmids #44025 and# 44026). Cells were transfected using a Nucleofector II system (Amaxa, Cologne, Germany), seeded at limiting dilution in 96-well plates, and selected for stably transfected clones in Tu2% medium containing puromycin (1 μg/mL). To identify clones containing the desired integrated GFP repair cassettes, 6 × 10^4^ cells were plated in individual wells of 12-well plates. Twenty-four h later, cells were transiently transfected with an I-SceI expression vector using Fugene 6 (11:1, Fugene 6: DNA) to generate double-strand DNA breaks. At 72 h post-transfection, cells expressing repair products were quantified by GFP positivity by flow cytometry using a BD FACSCanto II cytometer (BD Biosciences).

Intra-chromosomal reporter assays were carried out as described above for screening of stably transfected clones, except that cells were first infected with lentivirus vectors driving expression of either shScr or shNME1. Prior to infection, cells were seeded in 12-well plates (0.6 × 10^4^ cells/well) and allowed to attach for 24 h. Cell monolayers were then rinsed with PBS, followed by application of 0.3 mL of lentiviral supernatants. After 5 h, 0.6 mL of lentivirus dilution medium was added to the wells. Twenty-four h later, lentivirus-containing medium was removed, followed by maintenance of cells in Tu 2% medium for 48 h. Cells were then transfected with the I-SceI expression plasmid and subjected to flow cytometry as described above for the clone screening assay.

### 4.7. Statistical Analysis

ddI-PpoI qPCR assays were analyzed by ANOVA using SigmaPlot software. All data represent mean ± SEM of three independent replicate experiments. Significance was determined as *p* ≤ 0.05, indicated by asterisks (*). Intra-chromosomal assays of NHEJ, A-NHEJ and HR activity were analyzed by two-way ANOVA, with testing for main effects of replicate experiments and shRNA treatments followed by post-hoc comparisons of shScr versus shNME1 treatments by the Holm–Sidak method.

## Supplementary Materials

The following are available online at https://www.mdpi.com/1422-0067/21/16/5896/s1.

## Abbreviations

MSG	metastasis suppressor gene
NDPK	nucleoside diphosphate kinase
DSB	double-stranded DNA break
C-NHEJ	classical non-homologous end-joining
A-NHEJ	alternative NHEJ
HR	homologous recombination
NGS	normal goat serum
ChIP	chromatin immunoprecipitation
HA	hemagluttinin
ER	estrogen receptor
4-OHT	4-hydroxytamoxifen
DD	death domain
NER	nucleotide excision repair
RNR	ribonucleoside reductase
