# Supplementary material for: Metastasis Suppressor NME1 Modulates Choice of Double-Strand Break Repair Pathways in Melanoma Cells by Enhancing Alternative NHEJ while Inhibiting NHEJ and HR
Source: Int J Mol Sci. 2020 Aug 17;21(16):5896. doi: 10.3390/ijms21165896 (PMC7460576; doi:10.3390/ijms21165896)
Supplement: Supplementary file 1 [file ijms-21-05896-s001.pdf]

# Reduced expression of metastasis suppressor NME1 in melanoma cells promotes genomic stability by inhibiting alternative NHEJ and enhancing NHEJ and HR pathways of double-strand break repair

**Gemma Puts, Stuart Jarrett, Mary Leonard, Nicolette Matsangos, Devin Snyder, Ying Wang, Richard Vincent, Benjamin Portney, Rachel Abbotts, Lena McLaughlin, Michal Zalzman, Feyruz Rassool, and David Kaetzel**

## Supplementary Files

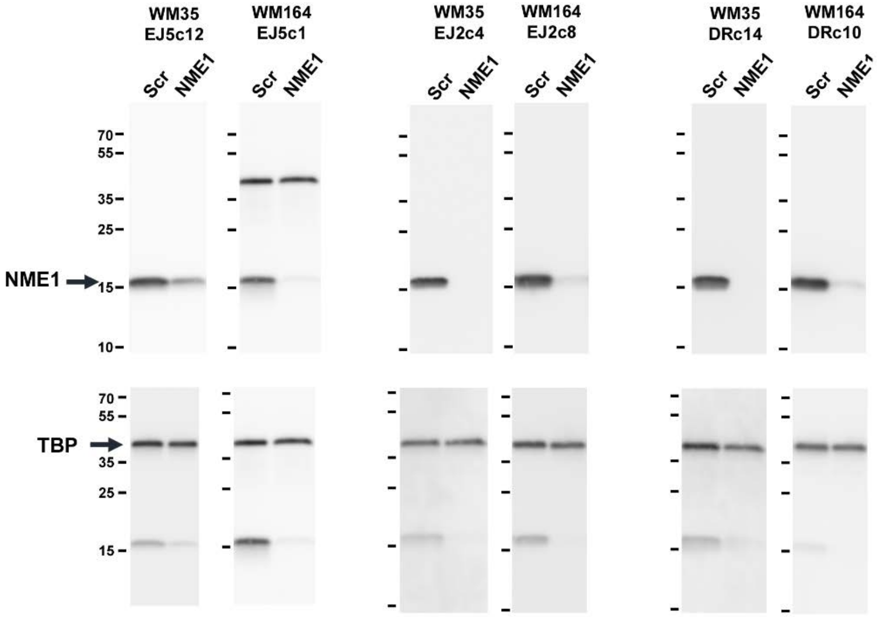


**Figure S1.** Full-length images of immunoblots shown in Figure 4.

**Table S1.** PCR Primers for I-PpoI assays.

| Primer set | Distance from I-PpoI target site (bp) | Sequence | Tm (°C) | Product length (bp) |
|---|---|---|---|---|
| 5 | $-3x10^6$ | 5′-CACCACGTCCAGCTGTTAAT-3′<br>3′-GGCCCTCACTCTGTCATTCT-5′ | 59 | 52 |
| 6 | -6280 | 5′-AAGTCCCTGCCCTTTGTACA-3′<br>3′-AGGGCCTCACTAAACCATCC-5′ | 59 | 64 |
| 7b | -3335 | 5′-CAAAGCGGGTGGTAAACTCC-3′<br>3′-TTCACGCCCTCTTGAACTCT-5′ | 59 | 114 |
| 8 | -2080 | 5′-CTGGGTATAGGGGCGAAAGA-3′<br>3′-GAGGGAAACTTCGGAGGGAA-5′ | 59 | 64 |
| 9 | -857 | 5′-GTAGGTAAGGGAAGTCGGCA-3′<br>3′-CAGCCCTTAGAGCCAATCCT-5′ | 59 | 64 |
| 10 | +19 | 5′-TTAGTGACGCGCATGAATGG-3′<br>3′-GCTGTGGTTTCGCTGGATAG-5′ | 59 | 69 |
| 11b | +572 | 5′-AGCAGGAGGTGTCAGAAAAGT-3′<br>3′-GCTTGGCGAATTCTGCTTCA-5′ | 59 | 128 |
| 12 | +1779 | 5′-GGAACTCCCTCTCCCACATT-3′<br>3′-GCAGCAGTGACTCCCTCTTA-5′ | 59 | 79 |
| 13f | +4515 | 5′-GCGATCTCAATTGCCTTTTAGC-3′<br>3′-TGACACAGAAGAACCCATGAGA-5′ | 59 | 75 |
| 14 | +6413 | 5′-TTGGGATCAGACTGGACCAC-3′<br>3′-ATACCCACGCACCCTTTACA-5′ | 59 | 101 |
| 15 | +43300 | 5′-AGCAGGTCTCCAAGGTGAAC-3′<br>3′-CTTGCCGACTTCCCTTACCT-5′ | 59 | 65 |
